# Therapeutic approaches to the treatment of recurrent respiratory papillomatosis of the aerodigestive tract (a clinical study)

**DOI:** 10.1080/13102818.2014.933500

**Published:** 2014-10-28

**Authors:** Toma Avramov, Evelina Vetckova, Maria Nikolova, Dinko Valev, Antoaneta Manolova, Maya Tafradgiiska, Dimitar Kostadinov, Ivan Tchalacov

**Affiliations:** ^a^ II ENT Clinic, University Hospital “Tzaritsa Yoanna”, Sofia, Bulgaria; ^b^ Bul-Bio-NCIPD Ltd., Sofia, Bulgaria; ^c^ National Reference Laboratory of Immunology, National Center of Infectious and Parasitic Diseases, Sofia, Bulgaria; ^d^ Bronchological Department, Specialized Hospital for Active Treatment of Pulmonary Diseases “St. Sofia” Ltd., Sofia, Bulgaria; ^e^ National Center for Public Health and Analysis, Sofia, Bulgaria

**Keywords:** recurrent respiratory papillomatosis, human papillomavirus, Calgevax, aerodigestive tract

## Abstract

Recurrent respiratory papillomatosis (RRP) is a rare disease, characterized by recurrent proliferation of benign squamous cell papillomas in the larynx as well as in the other parts of the aerodigestive tract. We have compared different treatment options for RRP of the aerodigestive tract including surgical, conservative and combined approaches. A total of 43 patients with papillomatosis that received a combined therapy were followed in the period from 2009 to 2013. The treatment included electrosurgery and CO_2_ laser surgery alongside with either immunotherapy with Bacillus Calmette-Guerin (BCG) (Calgevax) or α-interferon. In the control group without immunotherapy (*n* = 16) we used conventional microlaryngeal surgery. During the follow-up, relapse occurred in two patients for the CO_2_ laser surgery with Calgevax immunotherapy group (*n* = 16). In the group treated with α-interferon preceded by CO_2_ laser surgery (*n* = 9) and electrosurgery (*n* = 2), relapse had occurred in three patients. Among the control group, recurrence was observed in six patients. This required re-operation. Our data showed a three times more frequent relapses among patients who were operated with conventional surgery as compared to those operated with CO_2_ laser surgery and Calgevax immunotherapy, and two times more often relapses in patients operated with conventional surgery as compared to those with electrosurgery and CO_2_ laser surgery and application of α-interferon therapy. Conventional and laser surgeries have a palliative effect, though playing an important role in ensuring the airway patency. While specific antivirus treatment for human papilloma viruses does not exist, the immune modulation with Calgevax considerably reduces the frequency of relapses, by stimulating cellular immune effector mechanisms. The combined protocol allows rarefication of relapses and improvement of patients’ quality of life, but not complete healing.

## Introduction

Human papillomavirus (HPV) infection can cause formation of benign epithelial papillomas. The lesions were given their present name by Morell MacKenzie in 1871.[1] Recurrent respiratory papillomatosis (RRP) is a rare disease which is characterized by recurrent proliferation of benign squamous cell papillomas in the larynx as well as in the other parts of the aerodigestive tract.[2–4] Over 90% of the cases are associated with low-risk strains (types 6 and 11). However, RRP is a major clinical problem because of its location, often dramatic presentation due to significant airway obstruction, the ongoing resistance to therapies, high frequency of relapses, tendency of spreading to the lower respiratory tract and oesophagus (aggressive forms) and the possibility of malignant transformation into squamous cell carcinoma. A lot of lesions are asymptomatic at the beginning and are accidentally found during an examination or after a recent change in the voice and appearance of hoarseness. In practice, two types of papillomatosis are distinguished according to a number of clinical, histological and virusological peculiarities: juvenile (type I) and red recurrent papillomatosis in adults (type II). The trachea and the proximal bronchi are affected in only 5% of the patients and less than 1% have involvement of the disease in the lung parenchyma (mostly type I). In aggressive forms, the interval from primary affection of the larynx to spread in the lower airways is usually 12 years but in some patients this happens much faster. Although histologically benign, RRP is a serious clinical problem due to its location, resistance to therapies, frequent recurrences, spread in the lower airways and oesophagus(aggressive forms) and possibility for malignant transformation in squamous cell carcinoma.[5]

The most common current therapeutic approaches for treatment of RRP are surgical: microlaryngeal surgery (MLS) using either rigid or flexible bronchoscope; carbon dioxide (СО_2_) laser surgery; electrosurgery under bronchoscopic control and cryosurgery. MLS became an accepted surgical modality in the late 1950s and early 1960s, and since then has been used in procedures for functional improvement of the voice. СО_2_ laser laryngeal microsurgery still remains one of the main applications of the СО_2_ laser.[6–9] By means of flexible optical fibres or a rigid bronchoscope, laser energy can be brought to inaccessible areas (trachea and bronchi) and lesions are removed under optical control. The surgical treatment of the papillomatosis, which is still the principal treatment strategy,[5,10,11] aims at excision and histological examination of the proliferation; achievement of better functional postsurgical vocal result in case of laryngeal involvement and recanalization of the bronchial lumen. However, surgical methods have serious disadvantages including the lack of definite treatment results, high frequency of relapses and possible dissemination of the lesions during the intervention.[5] The palliative treatment effect of conventional and laser surgery has fostered the application of alternative treatment protocols. A number of centres have applied α-interferon therapy,[12–14] either separately or in combination with the above-mentioned surgical techniques.[2,6,13] Systemic (5 mg/kg i.v.) or local (intralesion injection under microscopic control) application of Cidofovir has also been reported [12] with yet inconclusive results. Recently, photodynamic therapy with /ALA/-5-amino-laevulinic acid has been used in patients with severe progressive forms of RRP affecting the larynx, tracheobronchial tree and oesophagus. This is a new option with good results but so far its application has been too limited to allow assessment of its efficacy.[2,12,15] The unsatisfactory results of conventional treatment protocols have prompted a possible association of frequent relapses with an inefficient antiviral immune response.[10] Control and resolution of viral infection require activation of natural cellular mechanisms combined with differentiation of interferon-γ, CD4 (Th1) and CD8 T cells, while a regulatory CD4+FoxP3+T subset prevents the antigen-specific clones from exhaustion, and potentiates formation of memory. Persistent infections are usually characterized with imbalanced differentiation of effector, memory and regulatory subsets.[16] The immunotherapeutic approach in RPR has recently gained an increasing interest. The Bulgarian immunomodulator Calgevax® (BCG) is a potent stimulator of Th1 response, with established therapeutic effect in superficial bladder carcinoma, and *melanoma malignum*.[17] Data about BCG effects in chronic HPV infection are limited.[18]

In this paper, we report the first results from a combined surgical and immunomodulatory treatment protocol with Calgevax® (BCG) in Bulgarian patients with RPR.[19] Our aim was to compare the efficiency of this approach with the established combined protocol surgery/α-interferon, and with surgical treatment alone.

## Materials and methods

During the period from 2009 to 2013, we have operated and followed up 43 patients with papillomatosis, who were either started on combined therapy or not. The patients were divided into three groups according to the applied treatment protocol: group І (*n* = 16) – CO_2_ laser surgery combined with BCG (Calgevax); group ІІ (*n* = 11) – comprised nine patients put through CO_2_ laser surgery combined with α-interferon and two patients subjected to electrosurgery combined with α-interferon; and control group ІІІ (*n* = 16) – conventional MLS. The average number of previous surgical interventions was 5.7 ± 1.6 with 6.37 ± 1.7 (group I), 5.45 ± 1.4 (group ІІ) and 5.19 ± 1 (group III).

The study met the ethical principles of the Helsinki Declaration, and was carried out after approval by the University hospital ‘Tsaritza Yoanna’ review committee; a written informed consent was obtained from all patients.

RRP was diagnosed using indirect laryngoscopy, flexible laryngoscope, rigid bronchoscope or microlaryngoscopy. All patients with papillomatosis were examined for accompanying lesions in the oral cavity, pharynx and oesophagus.[12,13] Surgical treatment of laryngeal and endobronchial papillomatosis was performed under endotracheal anaesthesia, during short apnoea or with high frequency jet ventilation, so as to achieve maximum visibility. The latter are recommended in the presence of disseminated papillomatosis. Papillary lesions undergo conventional MLS. Solitary papillomas which obturate segmental bronchi are removed with rigid bronchoscopy using endobronchial electrosurgery.[8] Excised lesions were subjected to pathohistological examination. Viral typing through polymerase chain reaction (PCR) was done in the Laboratory of Molecular Virology at the Military Medical Academy, Sofia.

Immune modulation with Calgevax® included 6–12 transdermal applications of 2.56 × 10^8^ clony forming unit (CFU) for the period of 45 ± 5 days, by scarification, without causing excessive bleeding, on an area of 25 сm^2^ from the patient's shoulder (local application of BCG is impossible due to the risk of oedema or laryngospasm). At the beginning we made 10 horizontal and 10 vertical lines on the skin of the first four patients. The following patients had 25 horizontal and 25 vertical lines at 2 mm and we observed better absorption of the medication in the skin.

α-Interferon was applied according to the following three-month scheme: 3 million IU (MIU), s.c., five times a week (Monday to Friday during the first month); 3 MIU, s.c., three times a week (Monday, Wednesday, Friday, during the second month) and finally during third month – 3 MIU, sub cutaneous (s.c.), once a week (Wednesday).

Parameters of cellular immunity (percentage and absolute counts of lymphocyte subsets) and cytokine expression were assessed in peripheral blood before and in the course of treatment to evaluate the effect from the immunotherapy.

## Results and discussion

The demographic characteristics of patients are given in Table 1.The age of the patients ranged from 2 to 66 years, and did not significantly differ between the groups (*p* > 0.05). Most of the patients (62.8%) were below 35 years of age. The average weight of patients was 72.84 kg, ranged from 13 to 123 kg and did not differ significantly between the studied groups (*p* > 0.05). The adult patients had various occupations (managers, economists, computer operators, students, accountants, retired, unemployed, etc.), with no evidence for association between occupational hazards and the onset of disease, or specific trends in the separate groups.

**Table 1.** Characteristics of the studied patients.

Characteristics	Group I	Group II	Controls
Number	16	11	16
Age (years)	36.13 ± 11.57	31.36 ± 18.35	31.19 ± 14.21
Sex (M/F)	10/6	7/4	8/8
Number of previous surgical interventions	6.4 ± 1.7	5.5 ± 1.4	5.2 ± 1.6
			
HPV subtype (PCR results)			
· HPV 6	11	8	11
· HPV11	2	1	2
· HPV 6/11	3	2	3
			
Localization			
· True vocal cords, bilaterally	7	5	8
· Glottis and supraglottis	3	2	3
· Glottis and subglottis	4	1	4
· Whole larynx	2	1	1
· Larynx and the trachea	–	1	–
· Tracheobronchial tree	–	1	–
			
Time of follow-up in months	36	45	48
Number of relapses	2	3	6
Time to relapse in months	4.5	3	1

**Figure 1.**
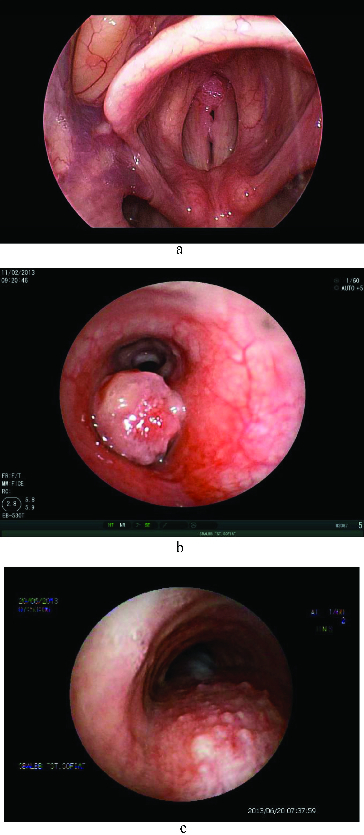
(a) Laryngeal papillomatosis. (b) Endobronchial solitary papilloma. (c) Tracheal papillomatosis.

HPV6 was detected in 18 cases (69.2%), HPV11 in three (11.5%), and five patients (19.2%) were diagnosed with mixed infection (HPV6/HPV11). Among the control group patients, HPV6 was detected in 11 cases (68.75%), HPV11 in two (12.5%), and three patients (18.75%) had a mixed infection (HPV6/HPV11). Again, significant differences between the groups were not detected. The following locations of the lesions were observed: true vocal cords – bilaterally (*n* = 20, seven patients on Calgevax treatment; five on interferon; eight with MLS) (Figure 1(a)), glottis and supraglottis (*n* = 8, three on Calgevac; two on interferon; three with MLS), glottis and subglottis (*n* = 9, four on Calgevax; one on Interferon; four with MLS), the whole larynx (*n* = 4, two on Calgevax; one on Interferon; one with MLS), larynx and trachea (*n* = 1, one patient on interferon) (Figure 1(b)) and the tracheobronchial tree (*n* = 1, one patient on interferon) (Figure 1(c)).

Patients in the CO_2_ laser surgery with BCG immunotherapy group (group I) were followed for an average of 36 months. Patients were monitored every three months. During the follow-up period, out of the 16 patients 10 had a strict compliance for control visits. We did not observe side effects or complications related to the treatment with Calgevax. A total of 14 out of the 16 patients strictly attended the control check-ups with a period of 45 ± 5 days between visits. Relapse occurred in two patients during the follow-up period between the fourth and fifth months after surgery. Control viral tests were performed in only three patients, but the results did not differ from the previous ones.

Patients from group ІІ (CO_2_ laser/electrosurgery combined with α-interferon application) were followed up for an average of 45 months. Control visits followed the same plan as for group I. During the follow-up period, 9 out of the 11 patients kept a strict compliance to the control examinations. Relapses were registered in three patients, 3 months after the operation. In this group, we observed some of the established side effects of α-interferon medication: fever, chills and fatigue. Side effects were seen in five of the patients from this group as we observed mild to moderate fever, chills and fatigue that responded well to medication with 500 mg paracetamol or 200 mg Nurofen and were resolved in the next 2–3 hours. Slight fatigue was observed by us during a three-month course in two of the patients.

Patients from the control group were followed up for an average of 48 months. Relapses were registered in six patients, one month after the operation on the average. The characteristics of the relapse did not differ between the three groups. Relapses imposed re-operation in all patients.

**Figure 2.**
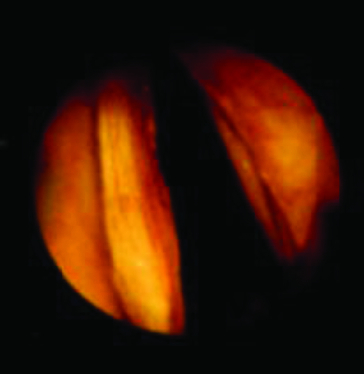
Six months after surgery.

To our knowledge, the current study publishes for the first time data concerning Calgevax immunotherapy applied after surgical treatment of RPR patients. According to our results, patients treated with conventional surgery had much more frequent relapses that occurred significantly faster as compared to those treated with combined CO_2_ laser (electro) surgery and immunotherapy. The relapses were twice more frequent as compared to combined α-interferon therapy, and three times more frequent as compared to combined ‘Calgevax’ treatment. In addition, BCG immunomodulation produced no side effects, unlike the application of α-interferon. A significant improvement of the voice was observed only at the beginning of the disease (Figure 2).

These results strongly support the benefits from the application of Calgevax as combined immune modulation therapy for RPR patients. Presently, no specific antiviral treatment exists that would allow complete eradication of chronic HPV infection. Surgery remains the only currently approved therapeutic approach in RRP, though associated with a significant risk of permanent laryngeal damage, as a result of repeated debulking, as well as with important emotional and social burden. Transoral laser microsurgery, though sparing for the patients, does not reduce the number of relapses.[10] Controlled trials fail to provide sufficient evidence for reliable conclusions about the effectiveness of antivirals (ribavirin, cidofovir, indole-3-carbinol) as an adjuvant therapy. Adjuvant therapy with interferon-α, in line with our data, does not completely prevent the relapse in RRP.[13]

BCG is currently the most successful agent that is used for cancer immunotherapy. The intravesical BCG immunotherapy of superficial bladder cancer is considered a ‘golden standard’.[16,20,21] However, the exact mechanism of the beneficial antitumour effects from the appliance of BCG is not yet fully understood. It is suggested that BCG contributes to the strengthening of the cell immune response in a non-specific manner, by activation of macrophages and lymphocytes.[22] The limited experience with BCG therapy in laryngeal carcinoma patients shows increased TNF-α and IL-6 secretion by macrophages as well as activation of the cytotoxic T lymphocytes with NK (CD16+CD56+CD3-phenotype).[18]

The recurrence in two of the treated cases showed that BCG-mediated immune modulation may not be efficient in every case and at any location. Further experience and longer follow-ups are warranted to precise the options and define the right duration of supporting immune-based medication.

## Conclusions

Conventional and laser surgeries have a palliative effect, though playing an important role in ensuring the airway patency. Our results show that antiviral treatment to allow general healing of the infections caused by HPV is not present. Various antiviral medications are effective for certain patients but have no effect to others and do not lead to substantial difference in the clinical manifestations of the disease. The different types of lesions and lesions in different anatomical areas require different methods of treatment. While specific antiviral treatment for HPV infections does not exist, the immune modulation with Calgevax considerably reduces the frequency of relapses, by stimulating the cellular immune effector mechanisms. The presented combined protocol allows rarefication of relapses and improvement of patients’ quality of life, but not a complete healing.
